# Far-Infrared Therapy Promotes Nerve Repair following End-to-End Neurorrhaphy in Rat Models of Sciatic Nerve Injury

**DOI:** 10.1155/2015/207245

**Published:** 2015-02-03

**Authors:** Tai-Yuan Chen, Yi-Chin Yang, Ya-Na Sha, Jiun-Rou Chou, Bai-Shuan Liu

**Affiliations:** ^1^Department of Radiology, Chi Mei Medical Center, Liouying, Tainan 736, Taiwan; ^2^Department of Neurosurgery, Taichung Veterans General Hospital, Taichung 40705, Taiwan; ^3^Department of Medical Imaging and Radiological Sciences, Central Taiwan University of Science and Technology, Taichung 40601, Taiwan

## Abstract

This study employed a rat model of sciatic nerve injury to investigate the effects of postoperative low-power far-infrared (FIR) radiation therapy on nerve repair following end-to-end neurorrhaphy. The rat models were divided into the following 3 groups: (1) nerve injury without FIR biostimulation (NI/sham group); (2) nerve injury with FIR biostimulation (NI/FIR group); and (3) noninjured controls (normal group). Walking-track analysis results showed that the NI/FIR group exhibited significantly higher sciatic functional indices at 8 weeks after surgery (*P* < 0.05) compared with the NI/sham group. The decreased expression of CD4 and CD8 in the NI/FIR group indicated that FIR irradiation modulated the inflammatory process during recovery. Compared with the NI/sham group, the NI/FIR group exhibited a significant reduction in muscle atrophy (*P* < 0.05). Furthermore, histomorphometric assessment indicated that the nerves regenerated more rapidly in the NI/FIR group than in the NI/sham group; furthermore, the NI/FIR group regenerated neural tissue over a larger area, as well as nerve fibers of greater diameter and with thicker myelin sheaths. Functional recovery, inflammatory response, muscular reinnervation, and histomorphometric assessment all indicated that FIR radiation therapy can accelerate nerve repair following end-to-end neurorrhaphy of the sciatic nerve.

## 1. Introduction

Numerous types of injury affecting the peripheral nervous system (PNS) [1] can lead to impaired nerve conduction and motor-sensory function, as well as altered skeletal muscle performance [2, 3]. Following injury in the PNS, the regular function of several components corresponding to the injured nerve undergo change; for example, an injured nerve can switch its role from relaying signals to growth and repair, or Schwann cells—which are responsible for myelination—start to degrade the myelin sheath. In addition to functional shifts, morphological changes, such as retrograde degeneration in the neurons of an injured nerve, Wallerian degeneration at the distal stump, axonal sprout formation at the proximal stump, and denervation of the muscles, can also occur [4]. Following transection injury, a direct end-to-end epineural suture can be used to join the proximal and distal stumps. Although the PNS can regenerate crushed or severed axons to a certain degree, the process is typically slow and incomplete. Furthermore, temporary impaired sensory and motor function results in neuropathic pain and major social consequences. Consequently, therapeutic intervention to obtain timely and appropriate nerve regeneration is necessary for full recovery.

Despite considerable advances in microsurgical techniques, the functional outcomes of peripheral nerve repair are generally unsatisfactory. Thus, therapeutic intervention strategies, such as delivering growth factors, electrical stimulation, physical activity, and surgical repair, have been undertaken to facilitate fast and appropriate nerve regeneration [5–10]. Clinicians have focused on developing effective methods to promote nerve regeneration, target organ reinnervation, and restore function at the injury site. Numerous physical and neurotrophic factors as well as pharmaceutical drugs influence nerve regeneration. Physiotherapy typically involves using therapeutic instruments for regenerative purposes [11]. Various forms of external stimulation have been employed to accelerate the regeneration process, which subsequently accelerates functional recovery. Such techniques include applying electrical [12], ultrasonic [13], and low-level laser (LLL) stimuli [14].

Infrared radiation is a nonvisible electromagnetic wave with wavelengths that are longer than those of visible light. Based on the differences in wavelength, the International Commission on Illumination recommends dividing infrared radiation into the following 3 bands: (1) near-infrared radiation (0.7–1.4 *µ*m); (2) middle-infrared radiation (1.4–3 *µ*m); and (3) far-infrared (FIR) radiation (3–1000 *µ*m). Infrared radiation transfers energy to the surrounding tissues, and it can be perceived as heat through thermoreceptors in the surrounding skin [15, 16]. FIR radiation therapy involves using low-energy light emitted from an artificial radiator. Recent studies have indicated that FIR radiation therapy can improve the prognosis of cardiovascular diseases [17, 18]. Moreover, FIR radiation can improve microvascular blood flow and angiogenesis in animal models [19]. Lin et al. [20] showed that FIR radiation therapy improves access flow and patency of arteriovenous fistulas in hemodialysis patients. Although FIR irradiation technology has been widely applied in health promotion [21–23] and food preservation [24, 25], the exact mechanisms underlying the hyperthermic effects and biological activities of FIR irradiation remain unclear.

Based on a review of the literature, no study has examined the effects of FIR radiation therapy on peripheral nerve injury regeneration; therefore, this study employed a rat model of sciatic nerve injury to investigate the effects of postoperative treatment with low-power FIR biostimulation on nerve repair following end-to-end neurorrhaphy. FIR radiation therapy was administered using an FIR therapy unit to irradiate the surgical site transcutaneously for 30 min (5 times per week over 2 weeks, beginning after the second postoperative day). This paper presents postoperative observations, nerve function analysis results, gastrocnemius muscular atrophy evaluation, and histomorphometric assessment. The findings elucidate the effects of FIR radiation on the recovery of injured nerves following end-to-end neurorrhaphy. We believe that this research is of clinical value for developing strategies for treating nerve injury.

## 2. Materials and Methods

### 2.1. Study Animals

Prior to conducting this study, the animal-use protocol was reviewed and approved by the Ethics Committee for Animal Experiments at Central Taiwan University of Science and Technology (Taichung, Taiwan). Adult Sprague-Dawley rats weighing approximately 250 to 350 g (BioLASCO Taiwan Co., Ltd) were used in the experiments. The rats were housed 2 per cage under controlled temperature and humidity conditions with 12 h cycles of light and dark and free access to food and water. The rats were randomly divided into the following 3 groups: (1) nerve injury without FIR biostimulation (NI/sham group; *n* = 6); (2) nerve injury with FIR biostimulation (NI/FIR group; *n* = 6); and (3) noninjured controls (normal group; *n* = 6).

### 2.2. Surgical Procedure

**Figure 1:**
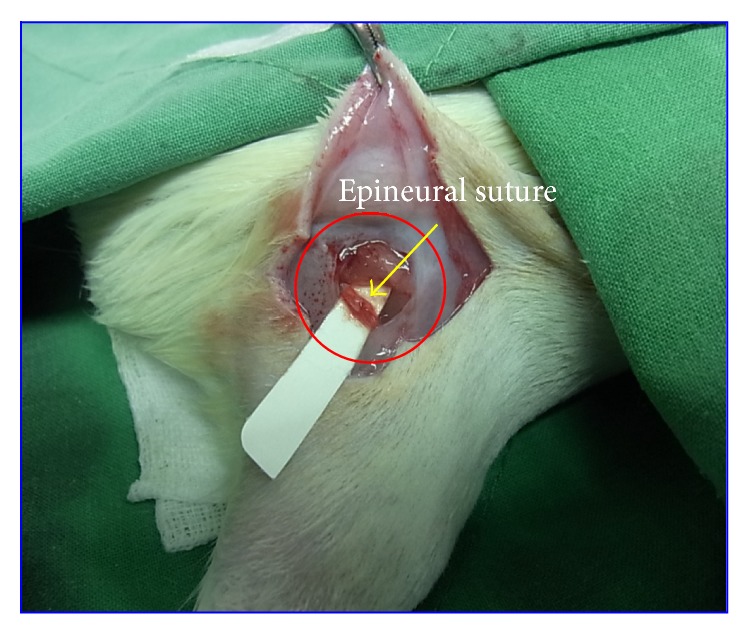
Photomicrograph of the surgical procedure. Right sciatic nerves were sectioned under germfree conditions and sutured through the epineurium.

The rats in all three groups were anesthetized using an inhalational anesthetic (VMS, Matrix, NY, USA). Anesthesia was induced using 4% isoflurane (Baxter, USA) and maintained using 2% isoflurane. The surgery area was shaved and then cleaned with 70% alcohol. Subsequently, an incision was made in the skin, and the fascia and muscle groups were separated through blunt dissection to expose the right sciatic nerve. In the normal group, the skin was closed using 2-0 silk sutures without further manipulation of the nerve. As shown in Figure 1, the right sciatic nerve of the NI/sham and NI/FIR rats was dissected from the middle region of the nerve; the distal stump was approached and sutured to the epineurium of the proximal stump. In all of the animals, the left contralateral sciatic nerve served as a positive control. The muscle layer was reapproximated using 4-0 chromic gut sutures, and the skin was closed using 2-0 silk sutures.

### 2.3. Far-Infrared Radiation Therapy

**Figure 2:**
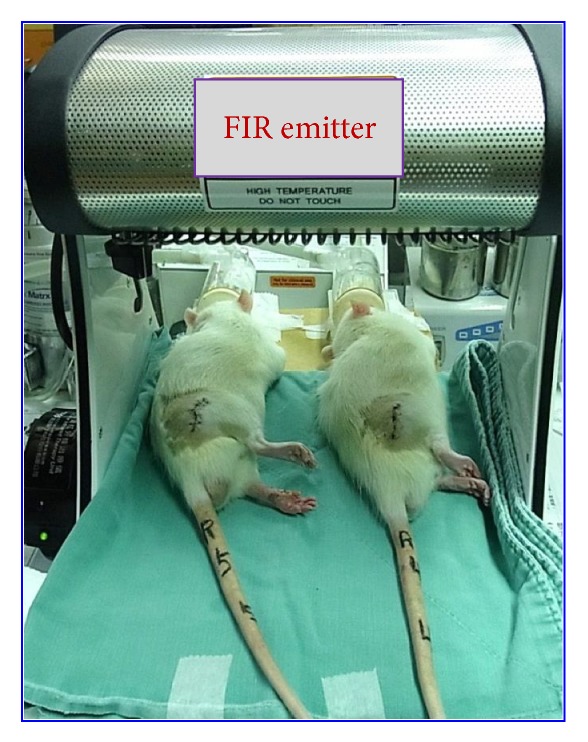
Experimental setup of the FIR radiation postconditioning tests. The device used was a WS TY101 FIR radiation emitter.

A TY301N therapy unit (WS Far-Infrared Medical Technology Co., Ltd., Taiwan) was used for the FIR therapy. To ensure that the abdominal skin temperature was slightly elevated, the top radiator of the unit was positioned 20 cm above the rats. The radiator used in this experiment could be adjusted manually or automatically to maintain a surface temperature between 38°C and 39°C. For the treatment group, the top radiator was positioned 20 cm above the anesthetized rats. The rats were irradiated for 30 min (5 times per week for 2 weeks, beginning after the second postoperative day).  Figure 2 shows the NI/FIR group undergoing FIR radiation therapy. The animals were handled gently, and the FIR biostimulation process did not produce any painful sensation or cause distress. The normal and NI/sham groups subjected to an identical procedure, but with the FIR switched off throughout the treatment.

### 2.4. Nerve Function Analysis

Nerve function recovery was assessed by calculating the sciatic functional index (SFI), as described by Bain et al. [26]. At predetermined times, the animals were examined to evaluate the recovery of nerve function by performing a walking-track analysis. All rats were trained to walk in a dark single-channel 100 × 7 cm closed box. Absorbent paper was placed on the floor of the box, and the rats' paws were dipped in ink before allowing them to walk in the box. Assessments were performed at weekly intervals following surgery. Measurements were recorded, and the SFI was calculated using the following formula:

(1) $$\begin{array}{c} \text{SFI}=\left(-38.3\times \text{PLF}\right)+\left(109.5\times \text{TSF}\right)+\left(13.3\times \text{ITF}\right)-8.8,\\ \text{PLF}=\frac{\left(\text{EPL}-\text{NPL}\right)}{\text{NPL}},\\ \text{TSF}=\frac{\left(\text{ETS}-\text{NTS}\right)}{\text{NTS}},\\ \text{ITF}=\frac{\left(\text{EIT}-\text{NIT}\right)}{\text{NIT}}, \end{array}$$

where print length (PL) denotes the distance from the heel to the tip of the third toe, intermediary toe spread (IT) indicates the distance between the first and the fifth toe, and toe spread (TS) is the distance from the second to the fourth toe (“E” and “N” indicate the treated and control or normal hind foot, resp.). Higher SFI values indicated higher degrees of functional recovery (a value of −100 represents total impairment).

### 2.5. Gastrocnemius Muscle Weight

To evaluate the reinnervation of the target muscle, the gastrocnemius muscles from both the operated and contralateral nonoperated limbs were dissected immediately prior to fixation perfusion. Subsequently, they were removed, dried on absorbent filter paper, and then weighed using an analytical balance. Briefly, both the left (nonoperated side) and right (operated side) gastrocnemius muscles were carefully cleaned and resected, dividing the tendinous origin and insertion from the bone by using an operating microscope. The muscles were weighed after harvesting. For each rat, the gastrocnemius muscle weight ratio (right/left, R/L) was calculated according to the weight of the gastrocnemius muscle on the operated leg (right leg) versus the normal leg (left leg).

### 2.6. Histological Assessment

Immediately after recording the walking-track analysis data, the gastrocnemius muscles and sciatic nerves were removed once the rats were sacrificed. The rats were anesthetized using 10% chloral hydrate (4 *μ*L/kg), were administered intraperitoneally, and were euthanized at 8 weeks after surgery. The sciatic nerves were exposed and carefully isolated from the surrounding tissue. Subsequently, the nerve segment was cut 4 mm proximal to the surgical site and 4 mm distal along the tibial and peroneal nerve branches. Sciatic nerve sections were obtained from the middle regions of the samples. The samples were immediately fixed in 4% paraformaldehyde for 24 h, then washed in 0.1 M phosphate-buffered saline (PBS), and divided into two segments to ensure precise localization. After fixation, some of the segments (gastrocnemius muscles and sciatic nerves: *n* = 6 in each group) were dehydrated in a graded ethanol series and finally embedded in Spurs' low viscosity resin. Subsequently, the embedded samples were cut at a thickness of 4 *µ*m by using a microtome with a dry glass knife and then stained with basic hematoxylin and eosin (H&E) stain. The sections were observed using an optical microscope (Eclipse E600, Nikon, CA, USA).

### 2.7. Immunohistochemical and Osmium Tetroxide Staining

The sciatic nerve slices were serially blocked with 0.3% H_2_O_2_/10% methanol in PBS for 10 min and 5% skim milk in PBS for 30 min. Subsequently, the nerves were incubated overnight at 4°C with primary antibodies used for CD4 (biossusa, 1 : 100) and CD8 (abcam, 1 : 100). Subsequently, the sections were rinsed with PBS and incubated with a SuperPicture polymer detection kit (Invitrogen) for 10 min at room temperature and then were rinsed 3 times with PBS for 2 min. Finally, the sections were visualized by color development with DAB enhancer and counterstained with hematoxylin. The immunostained sections were examined using the optical microscope. The other regenerated sciatic nerve tissue samples (*n* = 6 in each group) were fixed in 1% osmium tetroxide (Sigma, O5500, USA), dehydrated in a graded ethanol series and finally embedded in Spurs' low viscosity resin, cut transversely into 4 *μ*m thick sections, and stained with 0.1% toluidine blue after the operation. A blinded observer photographed sections of the regenerating bridge (approximately at its center) using a high-power optical microscope with a 400x zoom. Photographs were captured for each section used in the final quantitative analysis. Counting the nerve and fibers size, at least 30–50% of the sciatic nerve section area randomly selected from each nerve specimen, which was performed using Image-Pro Plus (Media Cybernetics, Inc., USA).

### 2.8. Statistical Analysis

In this paper, all numerical data are presented as the mean ± standard deviation. The differences among the results obtained under various testing conditions were evaluated using the one-way analysis of variance (ANOVA). The level of statistical significance was set at *P* < 0.05.

## 3. Results

### 3.1. General Observations after Operation

The animal models in this study tolerated the anesthetic and surgical procedures with no sign of infection. The animal models exhibited no complications typically associated with this type of surgical procedure, and all wounds healed without complication. Moreover, no signs of discomfort were observed throughout the 8-week evaluation period. All animals in the control and experimental groups survived the experimental period and exhibited normal eating and drinking behavior.  Table 1 presents the weight-change percentages of experimental rats during the surgical periods. A trend of increased weight-change percentages was observed in all groups during the postoperative surgical period. The weight-change percentages were all slightly higher in the FIR-treated group than in the NI/sham group, and a significant difference was observed at Week 8 (*P* < 0.05). We observed that FIR irradiation caused the wound healing at the site of the sutured skin to heal faster in the NI/FIR group compared with the NI/sham group at Week 1 after surgery (data no shown). Moreover, the FIR irradiation caused denser hair regrowth of glossier appearance in the FIR-treated rats than that in the NI/sham rats at 6 weeks after operation (data not shown).

### 3.2. Sciatic Nerve Function Recovery

The function of the gastrocnemius muscle was evaluated by performing a walking-track analysis.  Table 2 shows the functional assessment of posttraumatic sciatic nerve recovery based on the SFI. Normal walking is indicated before surgery where the SFI values are close to 0. As anticipated, both experimental groups (i.e., NI/sham and NI/FIR) exhibited a decline in SFI following surgery (*P* < 0.05). The SFI values increased considerably over time, and a similar trend of postoperative improvement was observed in both the NI/sham and NI/FIR groups. The mean SFI of the NI/FIR group was higher than the mean SFI of the NI/sham group in each period after surgery. The walking-track analysis yielded significantly higher SFI values in the NI/FIR group than in the NI/sham group after 4 weeks (*P* < 0.05). By Week 8 after surgery, the experimental group SFIs were approximately −71.43 ± 1.90 (NI/sham group) and −66.91 ± 2.69 (NI/FIR group), indicating significantly slower nerve recovery in the NI/sham group than in the NI/FIR group (*P* < 0.05).

### 3.3. Recovery of Gastrocnemius Muscle

**Figure 3:**
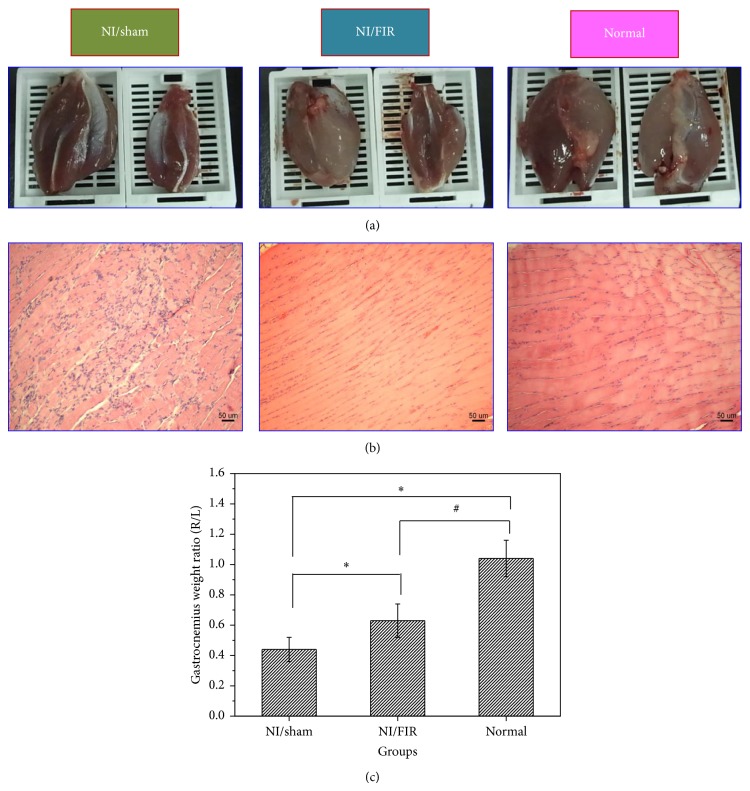
(a) Photographs of muscle atrophy at Week 8 following end-to-end neurorrhaphy (experimental muscles are on the right, and contralateral muscles are on the left); (b) light micrographs of transverse-sectioned gastrocnemius muscle with H&E staining; (c) gastrocnemius muscle weight ratios (right/left, R/L) in all groups at 8 weeks after surgery. ∗ significantly greater than the NI/sham group (*P* < 0.05); # significantly greater than the NI/FIR group (*P* < 0.05).

Following sciatic nerve injury, the innervation of the gastrocnemius ceased and progressive atrophy was initiated. Recovery of the gastrocnemius muscle following sciatic nerve injury and repair is expressed as a ratio between the ipsilateral and contralateral muscle weights.  Figure 3(a) shows that atrophy of the gastrocnemius muscle occurred 8 weeks following nerve injury (experimental and contralateral muscles are shown on the right and left, resp.). We observed partial atrophy of the gastrocnemius muscles, which was the target muscle of the injured donor nerve in both the NI/sham and NI/FIR groups. Atrophy of the donor muscle likely occurred because of the duration of denervation before reinnervation was fully achieved.  Figure 3(b) depicts sections from gastrocnemius muscles stained with H&E. Muscle fiber atrophy was observed in the NI/sham (left) and NI/FIR (middle) groups in comparison with the normal group (right). All groups maintained polygonal muscle fibers. The NI/sham group exhibited disseminated myofibril atrophy, cell denaturation, and necrosis, as well as inflammatory cell infiltration and proliferation. However, the control biopsy specimens that were removed from the NI/FIR and normal groups exhibited unremarkable pathological changes according to the H&E staining, and the ultrastructure of myofibrils was clear and orderly when observed with the optical microscope. The sciatic nerve injury caused a reduction in neural innervation of the gastrocnemius muscle, leading to a decrease in muscle weight. As shown in Figure 3(c), we quantified this reduction by weighing the excised gastrocnemius muscle and calculating the muscle weight ratio (denoted as R/L). The R/L ratio was obtained by dividing the weight of the experimental muscle (right) with that of the control muscle (left). As the R/L ratio approached a value of 1, we observed a reduction in atrophy. At Week 8 after surgery, the R/L ratios in the NI/sham, NI/FIR, and normal groups were 0.44 ± 0.08, 0.63 ± 0.11, and 1.04 ± 0.12, respectively. The statistical analysis results indicated significant differences between the NI/FIR and normal groups (*P* < 0.05), both of which were significantly higher in the NI/sham group (*P* < 0.05).

### 3.4. CD4 and CD8 Expression after Sciatic Nerve Injury

**Figure 4:**
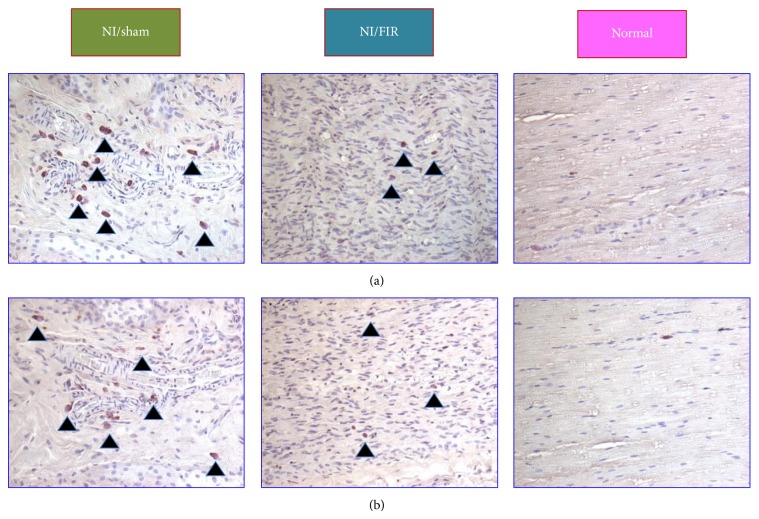
Inflammatory responses of injured sciatic nerves subjected to FIR radiation therapy.

Sutures and biomaterials can induce inflammatory cell deposits, which subsequently causes Schwann cell apoptosis. The severity of inflammatory cell deposits indicates the degree of inflammatory cytokine expression as well as the severity of nerve injury. The reduced cell apoptosis in the injured nerve is an indicator of nerve regeneration [27]. The number of accumulated inflammatory cells, such as macrophages and neutrophil, indicates the severity of local inflammation.  Figure 4 presents CD4 and CD8 antigen expression of the sciatic nerve at Week 8 after surgery. In the lesioned nerves, inflammatory cell recruitment first involved the resection site, where strong expression of CD4 (Figure 4(a)) and CD8 (Figure 4(b)) immunoreactivity was observed following end-to-end neurorrhaphy. A higher level of CD4 and CD8 antigen expression was observed in the NI/sham compared with that in the NI/FIR group. In the normal group, only minor CD4 and CD8 antigen expression was observed on some spindle-shaped cells resembling resident PNS macrophages.

### 3.5. Macroscopic Observation of Sciatic Nerve Regeneration

**Figure 5:**
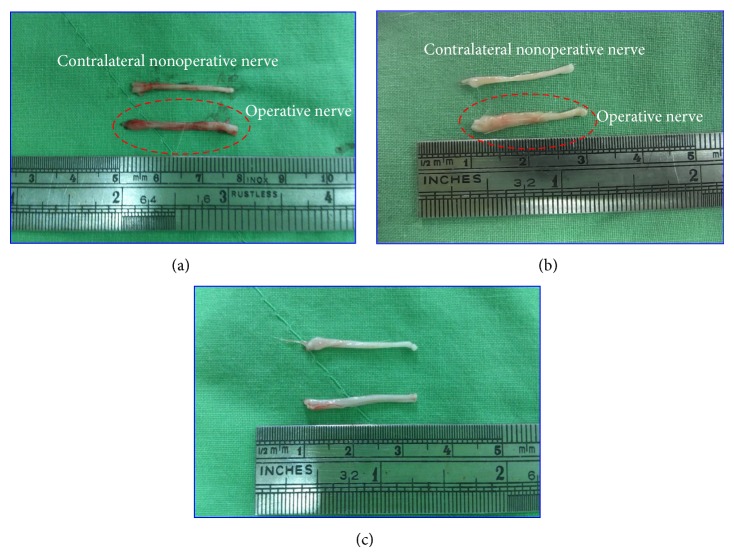
Photographs of the sciatic nerve of an adult rat at 8 weeks following end-to-end neurorrhaphy. (a) NI/sham group; (b) NI/FIR group; (c) normal group.

Figure 5 presents macrographs of the sciatic nerve at Week 8 after surgery. The sciatic nerve regeneration results showed that the nerve tissues in the NI/sham group (Figure 5(a)) were similar to those in the normal group (Figure 5(c)), although both were inferior to the NI/FIR group (Figure 5(b)). After surgery, the NI/FIR group exhibited more extensive axonal growth than did the normal and NI/sham groups. The results indicated that FIR radiation therapy promoted nerve regeneration and reduced the recovery time.

### 3.6. Histology and Remyelination of Regenerated Nerves

**Figure 6:**
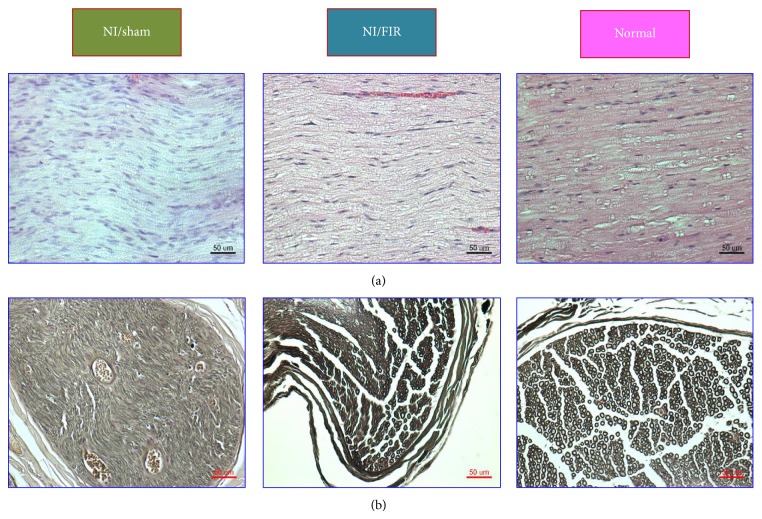
(a) Longitudinal sections with H&E staining; (b) transverse sections with osmium tetroxide of sciatic nerves from the medial segment of the regenerated nerve tissue (Week 8 after surgery) in all groups.

Figure 6(a) presents longitudinal sections of the regenerated nerve tissue of both the NI/sham and NI/FIR groups at Week 8 after surgery. The longitudinal sections at the injured site of the sciatic nerve were analyzed using H&E staining. Compared with the NI/sham group, the NI/FIR group exhibited linearly ordered structures and more compact histomorphological regeneration of nerve tissue. Moreover, the samples from the FIR-treated rats appeared more similar to the native nerve sample.

Figure 6(b) shows light micrographs of the transverse sections of regenerated nerve tissue of both the NI/sham and NI/FIR groups at Week 8 after surgery. Examination revealed a clear qualitative difference between the NI/sham and NI/FIR groups. The NI/sham group presented relatively low-density myelinated nerve fibers. By contrast, the NI/FIR group presented a higher density of myelinated nerve fibers in the regenerated nerve. In the NI/sham group, the untreated nerves were populated mainly by unmyelinated nerve fibers immersed in a collagen fibril-rich matrix, and evenly distributed intraneural blood vessels were observed in the regenerated nerve, indicating that angiogenesis had occurred during the nerve-regeneration process. However, numerous well-myelinated nerve fibers in the regenerated nerve tissue of the FIR-treated rats were distributed among numerous large fascicles. Qualitatively, sections from the NI/FIR group exhibited numerous large well-myelinated axons and histomorphological compaction. Both conditions were less-developed in the NI/sham group.  Table 3 shows quantitative analysis results comparing the regenerated nerve tissues of the NI/sham, NI/FIR, and normal groups. The morphological data of the regenerated nerve tissues in the NI/FIR and normal groups were significantly greater than that in the NI/sham group (*P* < 0.05), except for the axon diameter (compared with the NI/FIR group) and medial nerve area (compared with the normal group). Furthermore, the nerve fiber diameter and medial nerve area in the NI/FIR group were significantly greater than those in the normal group (*P* < 0.05). The quantitative results indicated superior nerve regeneration in the FIR-treated group compared with the NI/sham group. These results indicated that FIR radiation therapy accelerated and improved the regeneration of injured peripheral nerves in the rats.

## 4. Discussion

This study was conducted to determine whether FIR radiation influenced the regeneration of the sciatic nerve of rats subjected to injury through complete resection followed by epineural anastomosis and histomorphometric assessment. FIR is a potentially advantageous therapy because it requires only low-energy light emitted from a radiator; furthermore, irradiation of injured nerves requires no surgical intervention. Injury by complete resection was preferred to injury through crushing in this study. Crushing preserves the sustention structure of the nerve, because the neural tubes remain in continuity and axonal extension is increased, thereby facilitating regeneration. However, the principal aim of this study was to determine whether FIR radiation therapy influences nerve regeneration without interference or intervention. Various surgical modalities have been used in the repair of peripheral nerves, including epineural and perineural repair, autogenous grafts, vein grafts, and entubulation, with or without associated neurotrophic factors [28]. In this study, a simple epineural anastomosis method was adopted because of its easy implementation and because it demonstrates high biomechanical resistance to traction [29].

Previous animal and clinical studies have shown that thermal therapy, including FIR irradiation, can improve skin blood flow and reduce the frequency of some cardiovascular diseases [18, 30]. Vasodilatation caused by the thermal effect of FIR radiation therapy can increase microvascular blood flow and angiogenesis because of numerous energy transfers as deep as 2 to 3 cm in subcutaneous tissue. However, skin temperature was increased to only 38°C to 39°C after 30 to 60 min of FIR treatment as long as the distance from the skin was more than 20 cm. Therefore, infrared therapy can avoid the adverse effects of traditional thermal therapy methods, including burn injury, infection, and risk of access failure caused by prolonged compression. Other mechanisms that might explain the nonthermal effects of infrared therapy include the suppression of inflammation and improved endothelial function. A previous study demonstrated the biological effects of FIR on promoting skin wound healing with histological evidence of greater collagen regeneration and infiltration of fibroblasts expressing transforming growth factor-1 (TGF-1) in wounds [16]. Our results indicated that FIR irradiation promoted increased weight gain, regrowth of denser glossier hair (data not shown), and faster wound healing (data not shown).

SFI can be used to evaluate experimental results because of its high correlation with motor function recovery and morphological and morphometric regeneration of peripheral nerves following injury [31]. The SFI values obtained in this study indicated that severe function loss occurred in both experimental groups following nerve injury by complete transection; however, the mean SFI values increased over time, exhibiting a similar trend in postoperative improvement in both experimental groups, which indicated that some regenerated axons had passed through the injury site and eventually into the target organ. For this study, the SFI values of the NI/FIR group continually improved over time, reaching significantly higher levels than those of the NI/sham group at 4 weeks postsurgery (*P* < 0.05). FIR irradiation resulted in greater functional nerve recovery in the NI/FIR group (compared with the NI/sham group), indicating that FIR radiation therapy had a beneficial effect on the process of nerve regeneration over the evaluation period.

The gastrocnemius muscle is supplied by the posterior tibial branch of the sciatic nerve. Denervation of a target muscle occurs as a consequence of peripheral nerve injury, accompanied by a series of histological and biochemical alterations leading to final muscle atrophy [32]. If the muscle is reinnervated, muscle function is restored and atrophy ceases [33]. In this study, the gastrocnemius muscle weight recovered to 63% of the contralateral side in the NI/FIR group, and it was significantly higher than that observed in the NI/sham group (*P* < 0.05), indicating enhanced neural recovery and protection against muscle atrophy in the NI/FIR group. These data correlate with our SFI results, indicating that the gastrocnemius muscle weight recovery might be caused by appropriated reinnervation.

Nerve injury initiates an inflammatory response, which is essential for successful peripheral axon regeneration [34–36]. Traumatic injury to the PNS produces abrupt tissue damage at lesion sites. Nerve stumps located distal to lesion sites undergo Wallerian degeneration [36], which involves a complex series of molecular and cellular events that culminate in the recruitment of circulating proteins and white blood cells (leukocytes) to the injury site [34]. This influx of inflammatory cells triggers a series of nonneuronal cellular responses that lead to the clearing of debris in the peripheral nerve and production of an environment that is supportive of axon regrowth following injury [35]. Inflammatory cells and inflammatory mediators cause tissue damage and second-wave injury and play a role in the regeneration process. The attenuation of inflammatory cells, such as macrophages or neutrophil, rescues Schwann cells from apoptosis, which is related to enhanced nerve regeneration [27]. Both CD4 and CD8 antigens were originally described as antigen coreceptors on helper and cytotoxic/suppressor T lymphocytes, respectively [37]. Although macrophage expression of CD4 antigen is well-established [38], CD8 antigen has long been considered lymphocyte-specific [39]. Lin et al. [40] observed that FIR radiation therapy exerted a potent anti-inflammatory effect and induced HO-1 production. This study showed that FIR radiation therapy activated inflammatory factors in rat sciatic nerves following end-to-end neurorrhaphy. These results demonstrated the effectiveness and relevance of FIR irradiation in modulating the inflammatory process during recovery from sciatic nerve injury. The decreased expression of CD4 and CD8 in the NI/FIR group indicated that FIR modulated the inflammatory process during the recovery process.

Visual inspection after surgery indicated that the regenerated nerve tissue of the NI/FIR group was thicker than that of normal or NI/sham groups. In addition, the NI/FIR group exhibited more compaction, continuity, and comprehensive histomorphology in the regenerated nerve tissue in comparison with the NI/sham group. In transverse sections of the regenerated nerve tissue, the NI/FIR group exhibited neural tissue recovery over a greater area, as well as larger axon diameter and thicker myelin sheaths compared with the NI/sham group, indicating improved nerve regeneration. In this study, FIR radiation therapy on injured nerves modulated the inflammatory process and improved the capacity of myelin production. Thus, the effectiveness of FIR radiation therapy in promoting axonal growth in injured nerves was demonstrated in an animal model.

## 5. Conclusion

The findings of this study show that FIR radiation treatment is a novel and noninvasive therapeutic modality to improve motor function, accelerate recovery from sciatic denervation-induced gastrocnemius muscle atrophy, modulate the inflammatory process during sciatic nerve injury, and enhance nerve regeneration following end-to-end neurorrhaphy in a rat model of peripheral nerve injury. Future studies using FIR as a noninvasive treatment modality for various peripheral nerve diseases and injuries can lead to the wide acceptance and standardization of this innovative therapy in clinics.

## Figures and Tables

**Table 1:** Weight-change percentage of all rats during the experimental period.

Implantation time	NI/sham	NI/FIR	Normal
2 weeks	14.87 ± 4.10	15.58 ± 2.60	16.57 ± 1.41
4 weeks	16.92 ± 3.99	20.50 ± 1.38	20.52 ± 2.38
6 weeks	23.36 ± 4.90	29.13 ± 2.98	30.15 ± 3.65^*^
8 weeks	28.38 ± 3.24	36.88 ± 3.96^*^	36.29 ± 4.85^*^

^*^Significantly (*P* < 0.05) greater than NI/sham group at the same surgical time.

**Table 2:** Recovery levels of sciatic nerve function in all rats during the experimental period.

Weeks	NI/sham	NI/FIR	Normal
Before surgery	−7.33 ± 2.42	−7.08 ± 4.08	−6.34 ± 4.19
2	−84.78 ± 6.70	−78.61 ± 7.23	−6.81 ± 2.62^*^
4	−82.75 ± 6.18	−74.19 ± 2.38^#^	−7.47 ± 2.60^*^
6	−76.79 ± 3.36	−70.38 ± 3.82^#^	−8.11 ± 1.37^*^
8	−71.43 ± 1.90	−66.91 ± 2.69^#^	−7.62 ± 3.08^*^

^*^Significant differences between experimental (NI/sham and NI/FIR) and normal group at the same surgical time (*P* < 0.05).

^#^Significant differences between NI/sham and NI/FIR groups at the same surgical time (*P* < 0.05).

**Table 3:** Morphometric parameters of the regenerated nerve tissues at Week 8 after surgery in the NI/sham, NI/FIR, and normal groups.

Morphometric parameters	NI/sham	NI/FIR	Normal
Axon diameter (*μ*m)	3.19 ± 0.66	3.19 ± 0.45	6.42 ± 0.88^2,3^
Myelin sheath thickness (*μ*m)	1.72 ± 0.06	3.32 ± 0.27^1^	4.35 ± 0.37^2,3^
Nerve fiber diameter (*μ*m)	7.28 ± 0.45	9.82 ± 0.44^1,3^	8.19 ± 0.32^2^
Medial nerve area (*μ*m^2^)	501,633 ± 31,742	717,885 ± 13,940^1,3^	522,490 ± 7,258

^1^Significant differences between NI/sham and NI/FIR group (*P* < 0.05).

^2^Significant differences between NI/sham and normal groups (*P* < 0.05).

^3^Significantly differences between NI/FIR and normal groups (*P* < 0.05).
